# Leveraging a matrix of stakeholders to facilitate access to chemotherapy for women’s cancers in the Democratic Republic of the Congo

**DOI:** 10.3332/ecancer.2021.1234

**Published:** 2021-05-13

**Authors:** Tankoy Gombo YouYou, Kabongo Mukuta Mathieu, Michael L Hicks, Ronda Henry-Tillman, Alex Mutombo, Mukanya Mpalata Anaclet, Mulumba Kapuku Sylvain, Maya M Hicks, Leeya Pinder, Louis Kanda, Mirielle Kanda, Groesbeck P Parham

**Affiliations:** 1 Biamba Marie Mutombo Hospital, No. 9777, Boulevard Lumumba, Commune de Masina, Kinshasa, Democratic Republic of the Congo; 2 Department of Obstetrics and Gynaecology, University of North Carolina at Chapel Hill, 101 Manning Dr, Chapel Hill, NC 27514, USA; 3 Department of Obstetrics and Gynaecology, University Teaching Hospital – Women and Newborn Hospital, 10101 Nationalist Way, Lusaka, Zambia; 4 St Mary Mercy Cancer Center 36475 Five Mile Rd, Livonia, MI 48154, USA; 5 St Joseph Mercy Oakland Cancer Center 44405 Woodward Ave, Suite 202, Pontiac, MI 48324, USA; 6 McLaren Macomb Medical Center, 1000 Harrington Blvd, Mount Clemens, MI 48043, USA; 7 Winthrop P Rockefeller Cancer Institute, University of Arkansas for Medical Sciences, 4301 West Markham, Slot #725, Little Rock, AR 72205, USA; 8 Howard University College of Medicine, 520 W St NW, Washington, DC 20059, USA; 9 Department of Oncology, University of Washington, 1959 NE Pacific St, Seattle, Washington, DC 98195, USA; 10 Dikembe Mutombo Foundation, 400 Interstate N Pkwy, Suite 1040, Atlanta, GA 30339, USA; a https://orcid.org/0000-0002-1819-155X; b https://orcid.org/0000-0002-1782-9523; c https://orcid.org/0000-0002-1993-3367; d https://orcid.org/0000-0002-8929-7810; e https://orcid.org/0000-0001-5922-5990

**Keywords:** chemotherapy, South–south collaboration, Bioventures global health, Democratic Republic of the Congo, cervical cancer, breast cancer, Dikembe Mutombo Foundation, Biamba Marie Mutombo Hospital, fragile, conflict and violence-affected country

## Abstract

**Background:**

Cancer incidence is increasing worldwide. Over the next 20 years, the growing proportion of cases in low- and middle-income countries (LMICs) will account for an estimated 70% of all cancers diagnosed. The vast majority of cancer patients in LMICs will require chemotherapy, due to the advanced stage of their disease at the time of initial presentation. Unfortunately, the availability of cancer drugs in these environments is sparse, resulting in premature death and years of life lost. In an effort to lay a foundation for women’s cancer control in the Democratic Republic of the Congo (DRC), we implemented a programme which combined workforce development, infrastructure creation and cancer drug access. This manuscript reports on our experience with the latter.

**Methods:**

A private sector healthcare facility was selected as the programme implementation site. Workforce capacity was developed through a south–south partnership with an African national cancer centre. Cancer drugs were procured through a global cancer medicine access initiative.

**Results:**

A new chemotherapy infusion unit was successfully established at the Biamba Marie Mutombo Hospital in Kinshasa, DRC. A team of Congolese healthcare providers was trained at the Cancer Disease Hospital in Zambia to safely and effectively administer chemotherapy for breast and cervical cancer. Over 100 breast and cervical cancer patients have been treated with 337 courses of chemotherapy, without any serious adverse events.

**Conclusion:**

Common barriers to cancer drug access and its administration can be eliminated using regional educational resources to build oncologic workforce capacity, private sector healthcare facilities for infrastructure support and pharmaceutical consortiums to procure low-cost cancer medicines. By leveraging a matrix of global, regional and local stakeholders, the prevailing status quo of very limited access to chemotherapy for women’s cancers was creatively disrupted in DRC, Africa’s largest fragile, conflict and violence-affected country.

## Introduction

Cancer is on the rise in low- and middle-income countries (LMICs). It is predicted that without major interventions there will be a 70% increase in the disease burden by 2040 [1]. An estimated 60% of cancer cases and 75% of deaths will occur in LMICs [2]. In order to solve this looming public health crisis, the major pillars of cancer care—health promotion, vaccination, screening, early detection, diagnostics, treatment and palliation—must be implemented, widely disseminated and sustained, especially in low-resource settings where the disease burden is heaviest. Screening and early detection coupled with access to high-quality surgical, medical and radiation therapy have significantly improved disease outcomes in high-income countries [3]. Unfortunately, these services are extremely limited in LMICs, resulting in few opportunities for cure, shortened survival and little chance for palliation.

At the very core of the problem is a markedly insufficient cancer workforce (oncologists of all types, nurses with training in oncology, ancillary personnel, pathologists etc.), a weak diagnostic and therapeutic infrastructure, limited access to modern anti-cancer drugs and meagre health expenditure budgets [4–6], the latter often resulting in patients and their families bearing the burden of the high costs of diagnosis and treatment [7]. The number of oncologists in sub-Saharan Africa is scarce, [8] ranging from zero in countries like Lesotho, Benin, Gambia, South Sudan and Sierra Leone to only a handful in Malawi, Burkina Faso, Rwanda and Togo [9]. The average number of pathologists per capita in Africa is 1/1,000,000, in comparison to the ratio of 1/20,000 in the US and UK [10]. Basic infrastructure platforms (electricity, water, clinic space, operating theatres, equipment and supplies, reagents and ITC) are weak throughout sub-Saharan Africa; power and stock outages are not uncommon; and maintenance of equipment (CT scans, MRI machines, linear accelerators etc.) is minimal and unreliable, primarily because of the unaffordability of maintenance agreements [11].

Such is the case in the Democratic Republic of the Congo, a fragile, conflict and violence-affected nation whose population of 84 million people, >70% of whom live on less than $1.90/day, has no access to established cancer care facilities [12, 13]. Armed with previous experience in successfully establishing women’s cancer care services in highly resource-constrained African environments [14–19], we set out to disrupt the prevailing status quo and establish women’s cancer care services in a private sector healthcare facility (Biamba Marie Mutombo Hospital, BMMH) in the capital city of Kinshasa. Herein we describe the challenges and experiences of establishing the chemotherapy infusion unit. Other manuscripts in this series describe the overall effort as well as the specifics of establishing service platforms for cervical and breast cancer.

## Methods

### Workforce capacity development

#### Short course in chemotherapy infusion

The initial step in launching the chemotherapy infusion unit consisted of the education and certification of selected staff members from BMMH in the safe administration of cytotoxic chemotherapy agents. This included knowledge of the properties and classification of various drugs, their side effects, complications and safe administration, with an emphasis on the specific drugs used for breast and cervical cancer. To achieve this goal, an intense, hands-on, short course in chemotherapy administration was developed at Zambia’s national cancer centre—Cancer Diseases Hospital—in Lusaka, tailored for BMMH healthcare professionals. Under this curriculum, two BMMH general physicians and three nurses were trained to safely and effectively administer chemotherapy for breast and cervical cancer, following strict protocols. Prior to the beginning of the course, a pre-test was administered to the trainees to determine their baseline knowledge, followed by a post-test to measure the degree of mastery of the information that was taught, for purposes of validation and certification. Financial support was provided by the Howard G. Buffett (HGB) Foundation for all aspects of the short course, inclusive of international and local transportation, housing, a stipend, training fees, books and supplies. The didactic portion of the curriculum covered the following topics: treatment indications; classification and properties of chemotherapy agents; safe storage; mixing, transporting and disposal; proper dosing and safe administration; toxicities and potential complications; and management of complications. Hands-on training consisted of side-by-side observation and participation with Zambian clinical oncologists as they determined types and dosages of chemotherapy, pharmacists as they prepared the drugs for administration and nurse oncologists as they infused the drugs. The trainees also attended daily clinical rounds where they observed patients who were experiencing various drug-related adverse events. The educational curriculum was informed by multiple standard guidelines, inclusive of the National Comprehensive Cancer Network Harmonised Guidelines for sub-Saharan Africa [20]. The following modifications were made to further contextualise the guidelines for the conditions and circumstances (e.g., lack of radiation therapy) in the DRC: breast ultrasound in lieu of mammography for initial work-up; tamoxifen hormonal therapy provided for all breast cancer patients, in light of the absence of knowledge of tumour receptor status and no access to aromatase inhibitors or targeted therapy.

#### Safety protocols

A critical component of the short course curriculum focused on safety and included the following topics:

1. Personnel exposure—aerosol droplet and powder; dermal, oral and inhalation; food and water supply;
2. Patient safety—proper dosing, mixing and administration to prevent errors in administration and extravasation of vesicants;
3. Community safety—proper disposal and incineration of hazardous chemicals, adequate bathroom facilities in the chemotherapy unit, safe transportation of chemotherapy drugs and hazards waste through the hospital and proper storage of chemotherapy agents;
4. Management of complications—spills, extravasations, neutropenia, anaemia, excessive nausea and vomiting and dehydration.

Trainees were required to attain an 80% pass rate on the post-test for successful completion of the course.

### Infrastructure development

A previously unused room in the hospital was remodelled (painted, windows restructured to allow for ventilation etc.) and repurposed for chemotherapy infusion, including installation of an air conditioner, bathroom with toilets and sink for patients and a separate one for staff. A list of mandatory equipment and supplies was provided by the Cancer Diseases Hospital (CDH) staff in Zambia.

### Accessing cancer medicines

BMMH’s hospital and clinic records were used to predict the number of eligible breast and cervical cancer patients who would need drugs over the next 2 years. An agreement was made between the Dikembe Mutombo Foundation (DMF) and Bioventures Global Health (BVGH) [21] for the latter organisation to facilitate the acquisition of chemotherapy drugs through its *African Access Initiative.* BVGH facilitated purchase of the requested drugs from Novartis, a global healthcare company located in Switzerland, at an agreed upon price which included shipping to the DRC. Representatives from the two organisation (DMF and BMMH) worked together to attain approval of the Congolese government for importation of drugs into the country, and to ensure that the products shipped from Novartis cleared DRC customs without delay. Only agents approved by the United States Food and Drug Administration (USFDA) were accessed. BVGH communicated directly with Novartis, and DMF with DRC government officials and BMMH hospital administration. Product batches with at least 12 months of remaining shelf life were selected. Procurement documents (invoices and airway bills) were shared with the DMF at least 1 week prior to the anticipated arrival of products. Flights with arrival dates early in the week were selected to avoid storage of drugs in the airport over the weekend.

## Results

All Congolese trainees in the short course in Zambia were certified following passage of the post-test. Following educational activities in Zambia, the trainees returned to the DRC and organised the chemotherapy infusion unit at BMMH, under the virtual guidance and oversight of a clinical oncologist from the CDH. Once established, an oncology staff nurse from CDH visited the chemotherapy infusion unit at BMMH and for five consecutive days mentored and monitored Congolese staff as they administered cytotoxic chemotherapy agents to breast and cervical cancer patients. On the first day, problems were noted with potential personnel exposure related to improper use of eye protection and gowns during the mixing of chemotherapy, which was immediately corrected by the on-site oncology nurse mentor. Twelve patients received intravenous cytotoxic chemotherapy agents during the week of observation by the newly trained staff with no complications. Nausea and vomiting were managed appropriately with anti-emetics. There were no reported severe cases of neutropenia, thrombocytopenia, dehydration or the need for interim hospitalisation. The final assessment of the visiting CDH oncology nurse was that the newly trained staff were able to effectively and safely administer the required cancer medicines with only minor adjustments and corrections. Following hands-on mentoring and monitoring, safety and drug protocol issues were evaluated on a weekly then monthly basis via virtual audits x 6 months that were attended by a clinical oncologist from the CDH.

### Infrastructure development

All equipment and supplies recommended by CDH staff were successfully purchased with funds from the HGB Foundation (Figure 1). While the furniture was purchased locally, the HEPA mixing hood, refrigerator and all PPE were purchased from the US, as there were no vendors for such in the DRC. The HEPA mixing hood was installed to ensure the safety of personnel during the preparation of cytotoxic agents. Proper transport and disposal bins were also purchased locally and used exclusively for chemotherapy to ensure environmental safety.

### Access to cancer medicines

Cytotoxic chemotherapy for 500 courses/infusions of therapy for cervix cancer and 500 course/infusions for breast cancer were purchased. The average cost of infusion for chemotherapy at BMMH was $250–300/patient.

## Discussion

The obstacles to cancer treatment in sub-Saharan Africa are severe and multifaceted in nature. In a large survey of 31 cancer centres in Africa, those surveyed expressed concerns over the limited access to taxane chemotherapy and aromatase inhibitors, the absence of access to targeted therapy, the unavailability of radiation therapy and a lack of in-house pathology services [22]. Others have cited the lack of oncologic surgical capacity [8, 23].

To our knowledge, there were no clinical or medical oncologists within the country during the time of our training activities and programme implementation. This challenge alone can be a major limiting factor in establishing chemotherapy infusion services in LMICs. To overcome this barrier, we task-shifted the responsibilities of chemotherapy infusion to general physicians and nurses. This was facilitated using an abbreviated training course in chemotherapy infusion and a combination of virtual and hands-on mentoring and monitoring. Expert support for these activities was provided through a south–south collaboration with an established national cancer centre in the region.

Safety was of paramount importance during programme implementation and ongoing service provision. This was initially addressed through the short course curriculum. In addition, opportunities were created for an experienced oncology nurse from Zambia to be physically present in the newly established chemotherapy infusion unit in the DRC during start-up, and continued virtual monitoring thereafter. This allowed for close observation and immediate correction of any safety breeches. For further assurance, BMMH administration earmarked dedicated spaces for the warehousing, mixing and infusion of drugs and proper equipment, including personal protective equipment, biologic storage cabinets, biohazard transport and disposable bins.

To overcome the hurdles of hyperinflated prices for chemotherapeutic agents of unverifiable quality, we forged a relationship with BVGH through their *African Access Initiative* [21]. This allowed us to purchase drugs at reduced prices, thereby avoiding the excessively priced cancer medicines sold by private vendors in Kinshasa (3–5x’s the international price) and ensuring their quality as they were certified by the US Federal Drug Administration.

A previous attempt to build capacity for chemotherapy services in Africa through south–south collaborations involved the Ugandan Cancer Institute and the eSwatini Ministry of Health [24]. The goal was to establish a chemotherapy unit in eSwatini by training healthcare providers from eSwatini in Uganda. Unlike in our collaboration, there was no mention of certification and the training experience in Uganda was described as an observership programme, in contrast to ours which included hands-on training. Details of the challenges encountered, on-site mentoring and monitoring activities in Swaziland, construction of a chemotherapy infusion facility, how chemotherapy was accessed and establishment of safety standards were not mentioned.

### Limitations

A distinct obstacle that we initially encountered with this venture was the language barrier. The trainees were from a French-speaking African country, while the mentors were from the English-speaking nation of Zambia. All of the training material was initially written in English. We were able to overcome this barrier through translation of the written material into French and learning each other’s languages to the extent where basic ideas and concepts could be communicated. We feel this is a very important factor to report because language and ethnography might influence the inclusion of training candidates from non-English speaking countries.

The World Health Organisation has an Essential Medicines List that contains several chemotherapeutic and palliative agents used in the treatment and palliation of breast and cervical cancer. Other than tamoxifen, these medicines are difficult to acquire or are unaffordable for governments or patients in many sub-Saharan African countries. Purchasing chemotherapy, even at low cost, inevitably leads to resource limitations. However, if properly managed, it can be creatively leveraged to generate profit that can then be used to support future purchases.

## Conclusion

In June 2019, a chemotherapy unit was established at the Biamba Marie Mutombo Hospital in Kinshasa, DRC, in support of a broader effort to provide access to women’s cancer services. We leveraged the expertise of an established African cancer centre (Cancer Diseases Hospital of Zambia) to help train the local workforce, provide logistical guidance and oversight. Targeted investments in equipment and supplies along with modifications of pre-existing space inside a private sector hospital were used to create the necessary clinical infrastructure. Acquisition of high-quality chemotherapeutic agents at a reduced cost was facilitated through a novel global pharmaceutical consortium.

South–south collaborations are critical to building cancer care capacity in LMICs, as they can avoid some of the time-consuming and often wasteful missteps of north–south partnerships that may erroneously attempt to retrofit approaches that are not contextually appropriate for resource-constrained settings. Countries do not have to wait until all the pillars of cancer care—health promotion, prevention, detection, treatment and palliation—are adequately developed before sharing expertise. Instead, those that may have strengths in one particular area (e.g., surgical oncology) can share that expertise with another country in the region that has matched weaknesses. These partnerships can and should be designed, initiated, managed and controlled by leaders in the respective countries, to help ensure their relevance and impact.

## Abbreviations

BMMH: Biamba Marie Mutombo Hospital
BVGH: Bioventures for Global Health
HGB: Howard G. Buffett Foundation
CDH: Cancer Diseases Hospital
DMF: Dikembe Mutombo Foundation
DRC: Democratic Republic of the Congo

## Conflicts of interest

None of the authors declare any conflicts of interest.

## Funding statement

Funding for the initiative was provided by a generous grant from the Howard G. Buffett Foundation whose mission is to catalyse transformational change to improve the standard of living and quality of life, particularly for the world’s most impoverished and marginalised populations.

## Figures and Tables

**Figure. 1.** Chemotherapy infusion unit—equipment, supplies and structural modifications.

Remodelling of space for chemotherapy suite
HEPA mixing hood
Chemotherapy beds/chairs
Refrigerator (−20°C)
Personal Protective Equipment
IV Fluid, Tubing, Syringes, Needles and Tourniquets
Spill Kits
Chemotherapy drugs
Antiemetic agents
Chemotherapy transport and disposal bins
Protocols for administration of all drugs
Protocols for spills and extravasation
Protocols for the management of complications—neutropenia, anaemia, excessive nausea and vomiting and dehydration
